# A neuroscience-based approach to the assessment of sexual behavior in animals

**DOI:** 10.3389/fvets.2023.1136332

**Published:** 2023-04-04

**Authors:** Samet Tekin, Elif Ece Akgün, Ali Doğan Ömür

**Affiliations:** ^1^Department of Physiology, Faculty of Veterinary Medicine, Atatürk University, Erzurum, Türkiye; ^2^Department of Histology-Embryology, Faculty of Veterinary Medicine, Atatürk University, Erzurum, Türkiye; ^3^Department of Reproduction and Artificial Insemination, Faculty of Veterinary Medicine, Atatürk University, Erzurum, Türkiye

**Keywords:** hypothalamus, neuroscience, sexual behavior, photoperiod, reproductive physiology

## Abstract

Sexual behavior in animals is important in ensuring the continuity of the generation. These behaviors differ in animal species. Sexual behaviors are shaped under the control of the reproductive system. Physiological stimuli produced by the reproductive system find their counterparts in the organism as reproductive activity. Reproductive activity display a critical role by transferring on the genetic heritage of organisms to the next generations. This activity, which is built on delicate balances, is associated with many systems in the organism. Nervous system, hormonal system, and circulatory system are the main ones. The regular formation of the reproductive activity in species is due to the effect of various factors. In domestic mammals, the reproductive activity is regulated by hormones secreted from brain and endocrine glands. Many hormones have duties in terms of the sustainability of reproductive activity. GnRH is the main hormone responsible for initiating this reproductive activity. Gonadotropin-releasing hormone (GnRH), which is a small molecule peptide from certain nerve cells in the nucleus infundibularis region of the hypothalamus and consists of different amino acids, is secreted under the influence of smell, temperature, light, and physical stimulation. Besides, GnRH release is controlled by various neurotransmitters (adrenaline, noradrenaline, dopamine, acetylcholine, serotonin). On the other hand, various genetic factors in secretory glands, gonadal cells, reproductive tissues can lead to significant changes on reproductive activity through specific molecular pathways and mechanisms.

## Introduction

Reproduction is crucial for every animal species because it transfers genetic information to future generations. Reproduction in animals is under the influence of many factors. Environmental factors come first among these factors. When environmental factors become suitable, optimal conditions for reproductive activity are provided (1).

GnRH is produced in the hypothalamus and then secreted into the hypophyseal portal circulation. These gonadotropic cells secrete the reproductive hormones follicle stimulating hormone (FSH) and luteinizing hormone (LH) into the bloodstream (2). A glycoprotein hormone, LH is released by the gonadotrophin cells of the adenohypophysis in tandem with FSH. There is a neural circuit that includes the hypothalamus, pituitary gland, and testes that produces luteinizing hormone. While LH secretion is increased by gonadotropin-releasing hormone (GnRH), it is suppressed by estrogen in females and testosterone in males through this route (3). It is widely established that FSH plays a key role in folliculogenesis by stimulating the development of a big pre-ovulatory follicle that is capable of ovulating and developing a corpus luteum in response to the mid-cycle surge of LH (4).

In terms of reproductive physiology, there are two basic functional units in males and females, where the effectiveness of hormones is reflected: testis and ovary. The testis contains two main somatic cells, the Sertoli cell and the Leydig cell. Leydig cells are found in the intertubular/interstitial area of the testis, whereas Sertoli cells are confined to the tubular compartment and serve as the anchor for germ cell growth. Sertoli cells have functional FSH receptors and Leydig cells have LH receptors (5). The ovary is a crucial component of the female reproductive system. The ovary's primary tasks are the production and periodic release of oocytes and the secretion of steroid hormone (6).

Environmental factors are important in shaping sexual behaviors. One of the environmental factors is light. Appropriate and continuous light can stimulate reproductive activity in animals. Light stimulates the eye's photoreceptors, activating the reproductive axis. Photoreceptors can determine whether the rays change daily or seasonally and accordingly stimulate the reproductive axis (7). Animals with seasonal cyclic activity follow this seasonal light period.

Another environmental factor is odor. According to the characteristics of the smell taken, sexual behaviors are stimulated in animals. Pheromones that come out with odors are perceived by the vomeronasal organ located in the noses of animals. The process is shaped by some hormonal and neural stimuli that result in the emergence of sexual behaviors (8). On the other hand, there is a connection between temperature and reproductive parameters. Increasing temperature suppresses both sperm quality in male animals and ovarian activity in females through the hormonal mechanism (9).

### An important stage in the embryo: Sexual differentiation

Primary sex-biasing factors are encoded by sex chromosomes that differ in male and female zygotes. The transcriptome of XX and XY embryos becomes sexually differentiated at the 2–8 cell stage of division (10). The sex of embryonic cells can be assessed through gene expression (11).

The sexual differentiation of the brain and the behavior shaped by it largely occur perinatally in rodents and prenatally in primates, and this process is under the control of hormones. Sex programming of the brain by hormonal exposure in the early stages of life has been an area open to various researches. Sex-related differences in male and female brains are linked to epigenetic changes (12).

Regarding the epigenetic effect, in the rat model, inhibition of deacetylating enzymes in males during the perinatal critical period impaired the adult mating behavior of these animals. Epigenetic modifications of the aromatase gene-associated chromatin also strongly support the conclusion that it contributes to sex differences in expression and thus to estrogen production and masculinization (13).

On the other hand, when an evaluation is made in terms of neuroanatomical sex differences, the numbers of neurons, astrocytes and microglia are important. Depending on gender, neurons die as a direct result of steroid action or due to lack of trophic support by androgens or estrogens (14).

Regarding the sexual differentiation of the brain and its reflection on the behavior of the creature throughout its life, the human species, which cannot be independent of culture, socialism, emotions and prejudices, and on the other hand, creatures such as hermaphrodite worms, parthenogenic lizards, and sex-changing fish are not positive examples from which inference can be made (15).

**Figure 1:**
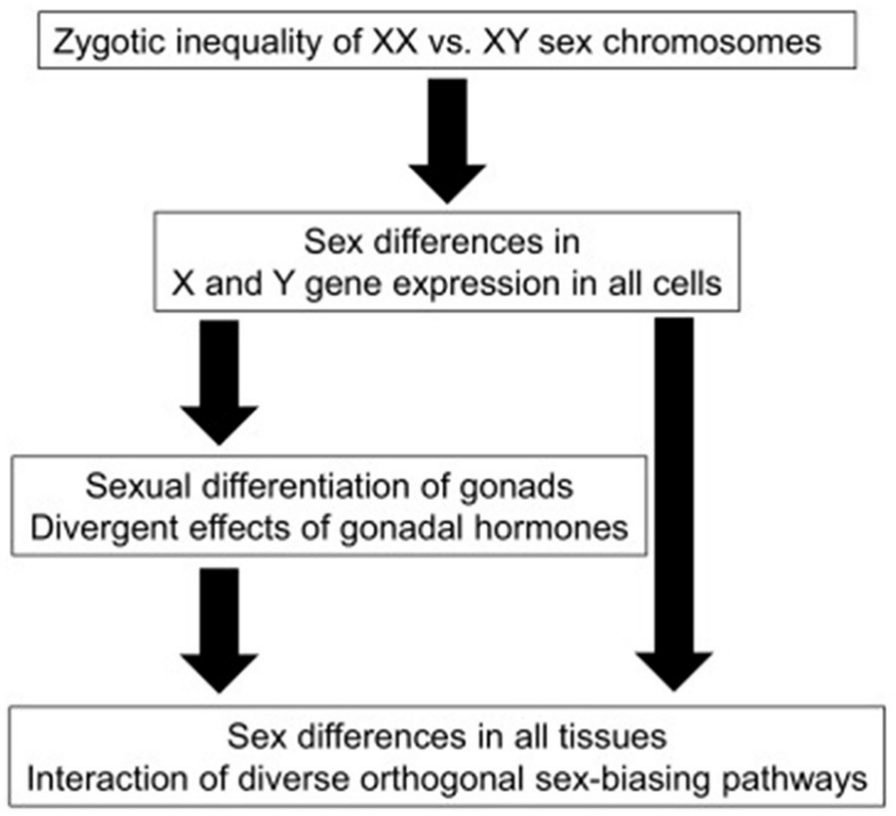
A schematic diagram illustrating sex-biasing influences of sex chromosomes and gonadal hormones (16).

As expressed in Figure 1, generally, in mammals, immediately after fertilization, if the Y chromosome is present, the X chromosome is inactivated. In the first weeks of embryological development, the embryo has Müller and Wolf ducts, the precursors of both female and male reproductive systems. In the following weeks, protein synthesis starts from the SRY (sex-determining region Y) gene on the Y chromosome and TDF (Testis-determining factor) protein is formed. In addition, the Histocompatibility Y antigen on the Y chromosome ensures the development of testis, Sertoli cells, and interstitial tissue in the embryonal-fetal period. During this period, Leydig cells form and begin to produce testosterone. Testosterone suppresses the Muller channel (AMF-SERTOLI) and supports the development of the Wolf channel. Wolf duct develops to form male genitalia. Through development of the testicles increases the secretion of testosterone and this accelerates the gaining of the male character of the embryo. If the individual does not have a Y chromosome, the Müllerian duct develops, in which case the embryo develops as a female. In some individuals, the SRY gene on the Y chromosome is faulty or cannot be expressed correctly, resulting in females with XY chromosomes. It is impossible to distinguish these individuals from normal females with their external appearance (17–20).

**Figure 2:**
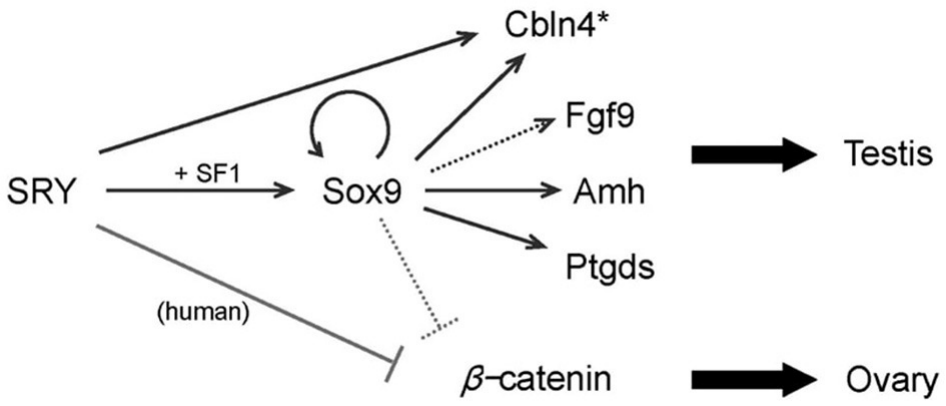
A model of SRY action in testis determination.

SRY upregulates Sox9 expression and then Sox9 activates several downstream genes such as Amh, Cbln4, Fgf9 and Ptgds. The orchestration of these genes promotes testis differentiation. SRY also inhibits β-catenin activity. At least in humans, the inhibition is mediated by the direct protein-protein interaction, whereas in mice it would be mediated *via* activating the downstream gene(s), presumably Sox9. The lines show direct transcriptional regulations and protein interactions, respectively (Figure 2). The broken lines indicate putative links. Asterisk, Cbln4 function for testis differentiation is unknown (18).

## Functional units that take an active role in reproduction

### Hypothalamus and pituitary gland

The hypothalamus is a little area in the middle of the brain that is made up of nerve cells and a collection of nuclei with many different roles. As a connecting structure between the neurological and endocrine systems, it plays a crucial role in regulating internal body temperature, or homeostasis (21). In the ventral brain, the hypothalamus is placed above the pituitary gland and below the third ventricle (22). The anterior part of the hypothalamus, above the optic chiasm, is known as the supraoptic area. It comprises the supraoptic, preoptic, and medial pre-optic nuclei, the suprachiasmatic and anterior hypothalamic nuclei, and the paraventricular nucleus (21). The posterior lobe comprises nerve terminals of hypothalamus-derived neurons. The pituitary stalk links the hypothalamus to the pituitary gland and transmits the hypothalamic axons to the posterior lobe, as well as regulating hormones from the hypothalamus to the anterior lobe through a portal system (23).

The hypothalamus secretes a variety of hormones into the bloodstream that eventually makes their way to the anterior pituitary; these include thyrotropin-releasing hormone (TRH), gonadotropin-releasing hormone (GnRH), growth hormone-releasing hormone (GHRH), corticotropin-releasing hormone (CRH), somatostatin and dopamine (22).

Vasopressin and oxytocin are two hormones produced in the hypothalamus that move directly to the posterior pituitary through hypothalamic neurons. Vasopressin, also known as antidiuretic hormone (ADH), is produced by the supraoptic nucleus and stored in the posterior pituitary lobe; it regulates blood pressure and the body's overall water balance (22). Oxytocin increases uterine contractions during delivery and milk production when a baby starts breastfeeding (24).

The pituitary gland is made up of two primary parts, the anterior lobe and the posterior lobe, and it is located in a depression in the sphenoid bone called the sella turcica in the median portion of the middle cranial fossa. The anterior lobe is divided into the pars distalis, pars intermedia, and pars tuberalis. The anterior pituitary's main gland, the pars distalis, is in charge of secreting hormones into the body's periphery (25). The anterior pituitary, known as the adenohypophysis, and the posterior pituitary, known as the neurohypophysis, are the two lobes that make up the pituitary gland, which serves as a crucial connection between the neurological and the endocrine system (26). In the anterior lobe (pars anterior) of the hypothalamus, called adenohypophysis is characterized by acini that are well-delineated and typically include a variety of hormone-producing cells. This nesting structure is most clearly seen in reticulin preparations, which outline the acini margins (27). There are no neuroendocrine epithelial cells present in the neurohypophysis. Instead, it is formed of axons emanating from clusters of hypothalamic neurons, most notably magnocellular neurons of the supraoptic and paraventricular nuclei. Their terminals finish close to the sinusoids of the posterior lobe, where they form the hypothalamo-hypophyseal tract (27).

**Figure 3:**
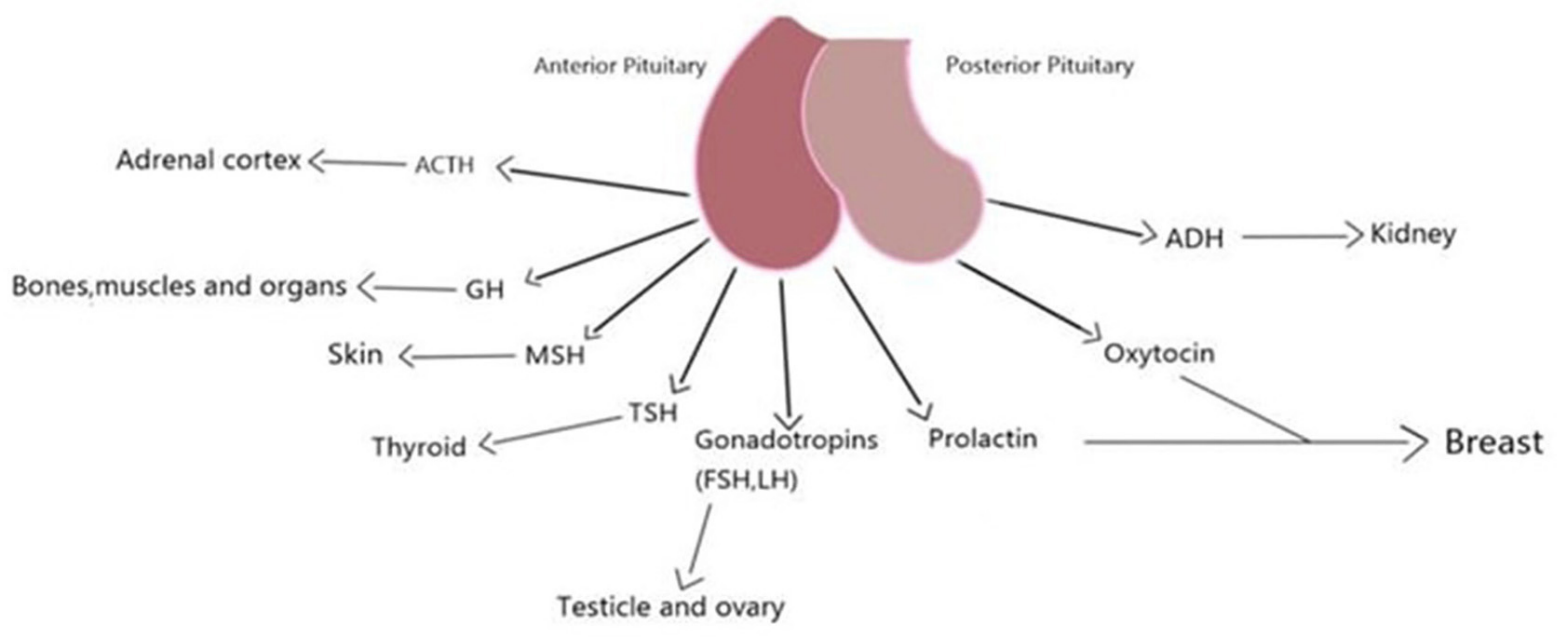
Pituitary, its secretions and the tissues it affects.

The five morphologically diverse endocrine cell types that make up the anterior pituitary are generated from the ectodermal cells in Rathke's pouch, and each type produces a different hormone. These endocrine cells mature in a certain order, starting with corticotropes, then thyrotropes, gonadotropes, somatotropes, and lastly lactotropes (26) (Figure 3). The anterior pituitary has many cell types that may be distinguished from one another based on their histology (28). The cytoplasmic staining abilities of the anterior pituitary's cells were the only criteria used to categorize them as chromophobe, acidophile, and basophile cells (26). While somatotroph and lactotroph cells could be stained acidophilic, corticotroph cells could be stained basophilic. Besides, gonadotroph and thyrotroph cells could be either basophilic or chromophobe (Gray's anatomy p380, 39th edition). About half of the epithelial cells in the anterior pituitary are chromophobe cells; these cells are tiny and do not respond to standard Staining. Degranulated secretory cells and stem cells are also included in this group (28). The anterior pituitary gland's star-shaped follicle-forming cells known as folliculo-stellate cells (FS-cells) were initially discovered by electron microscopy as non-endocrine agranular cells (29). FS-cells are small, chromophobic cells that stretch between the endocrine cells. These cells could be stained with S100 and GFAP antibodies, however, their expression patterns are not always similar and may indicate distinct developmental or functional phases (27). While chromophil and chromophobe cells generate hormones, follicular-stellate cells do not (28).

### Testes and ovarium

**Figure 4:**
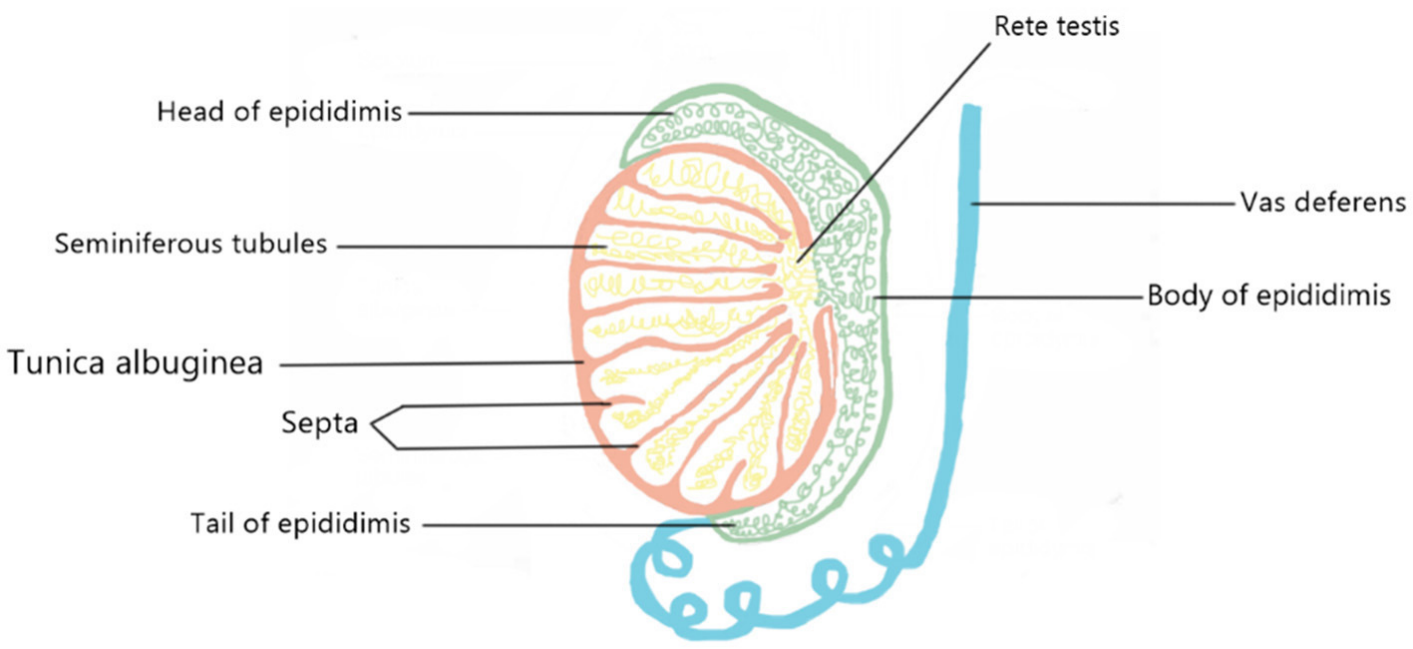
A schematic model of the appearance of the testicular tissue.

The parenchyma of the testes is encased in a capsule made up of three layers: the tunica vaginalis, the tunica albuginea, and the tunica vasculosa. Septa, tunica albuginea, separate the testicle into compartments. Each septum divides seminiferous tubules and interstitial tissue, which contains Leydig cells, blood vessels, lymphatics, mast cells, neurons, and macrophages. Seminiferous tubules consist of Sertoli, germ, and peritubular myoid cells. They loop tubules connected to the rete testis, a network of collecting tubes that combine to produce efferent ducts that transport testicular fluid and spermatozoa to the caput epididymis (30) (Figure 4).

Leydig and Sertoli cells are responsible for male reproduction. The interstitium of the testes contains Leydig cells, which are located close to the seminiferous tubules. The cytoplasm of these cells is pink, and a histological test for pink crystals of Reinke will confirm their identity (31). Sertoli cells place at the seminiferous tubules' basolateral aspect and may be recognized by their huge, irregularly shaped cells that line the inner perimeter and their smaller, more evenly formed circular neighbors (32). The sertoli cells have a role in both embryonic testis development and adult spermatogenesis by controlling the local microenvironment in which the germ cells are forming. They are positioned in a way that allows them to stay in close touch with the germ cells, separate the germ cell populations into their own microenvironments inside the seminiferous epithelium, and control the biochemical conditions in those microenvironments (33).

**Figure 5:**
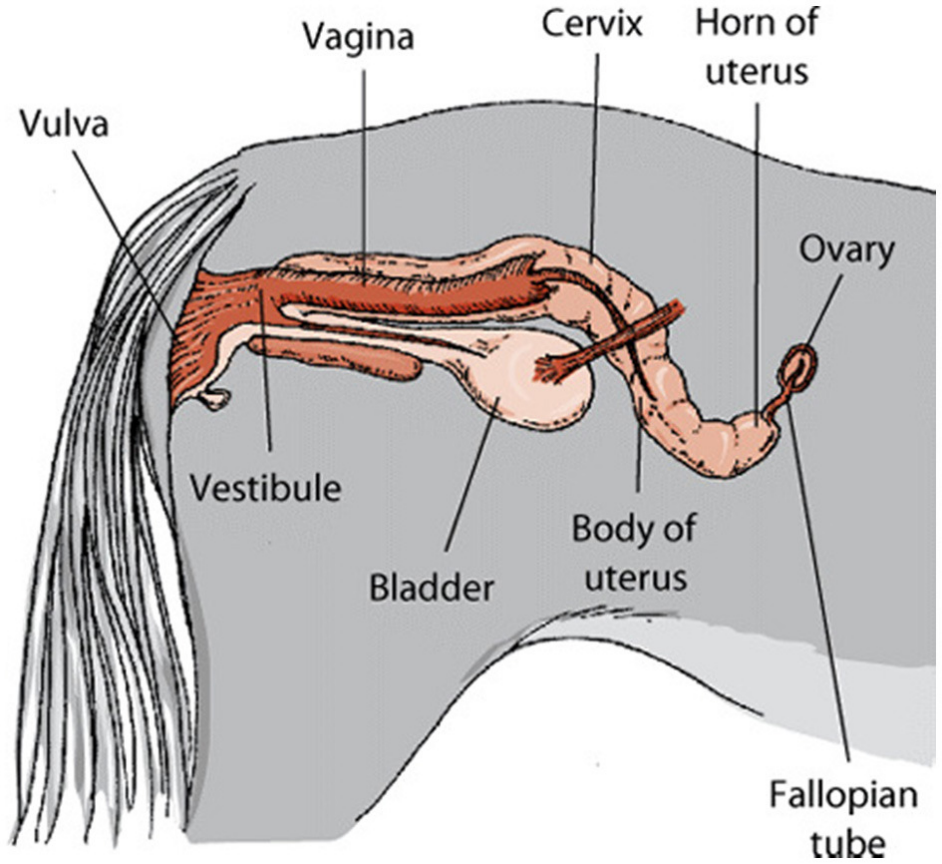
Genital system of mare.^1^

The ovaries are the gonads, or reproductive organs, that sit near the fimbriae at the end of the uterine tubes. Each one is about the size of an almond and is between 2 and 3 centimeters in length. The mesovarium, a double fold of peritoneum that makes up the wide ligament, serves as a structural foundation for the ovaries. The ovarian blood and lymph vessels are located in the peritoneum, also known as the suspensory ligament. The ovarian ligament connects the ovary to the uterus. The ovary has a cuboidal epithelial layer that is superficial to a tunica albuginea layer of thick connective tissue. The cortex, or interior, of the organ lies underneath its outermost layer, the tunica albuginea (34). Cattle and sheep have almond-shaped ovaries, but horses have bean-shaped ovaries due to the existence of a distinct ovulation fossa and a depression in the ovary's associated border (Figure 5). The ovary of a pig resembles a cluster of grapes because projecting follicles and corpora lutea hide the ovarian tissue underneath (35).

The outer epithelium of the ovary is termed the germinal epithelium, and it is composed of simple cuboidal cells. The tunica albuginea, a layer of collagenous connective tissue, lies underneath this one. Follicles may be found in the cortical layer of the ovary. Follicles of varying sizes and stages of development are seen here. The medulla is the central part of the ovary (36).

**Figure 6:**
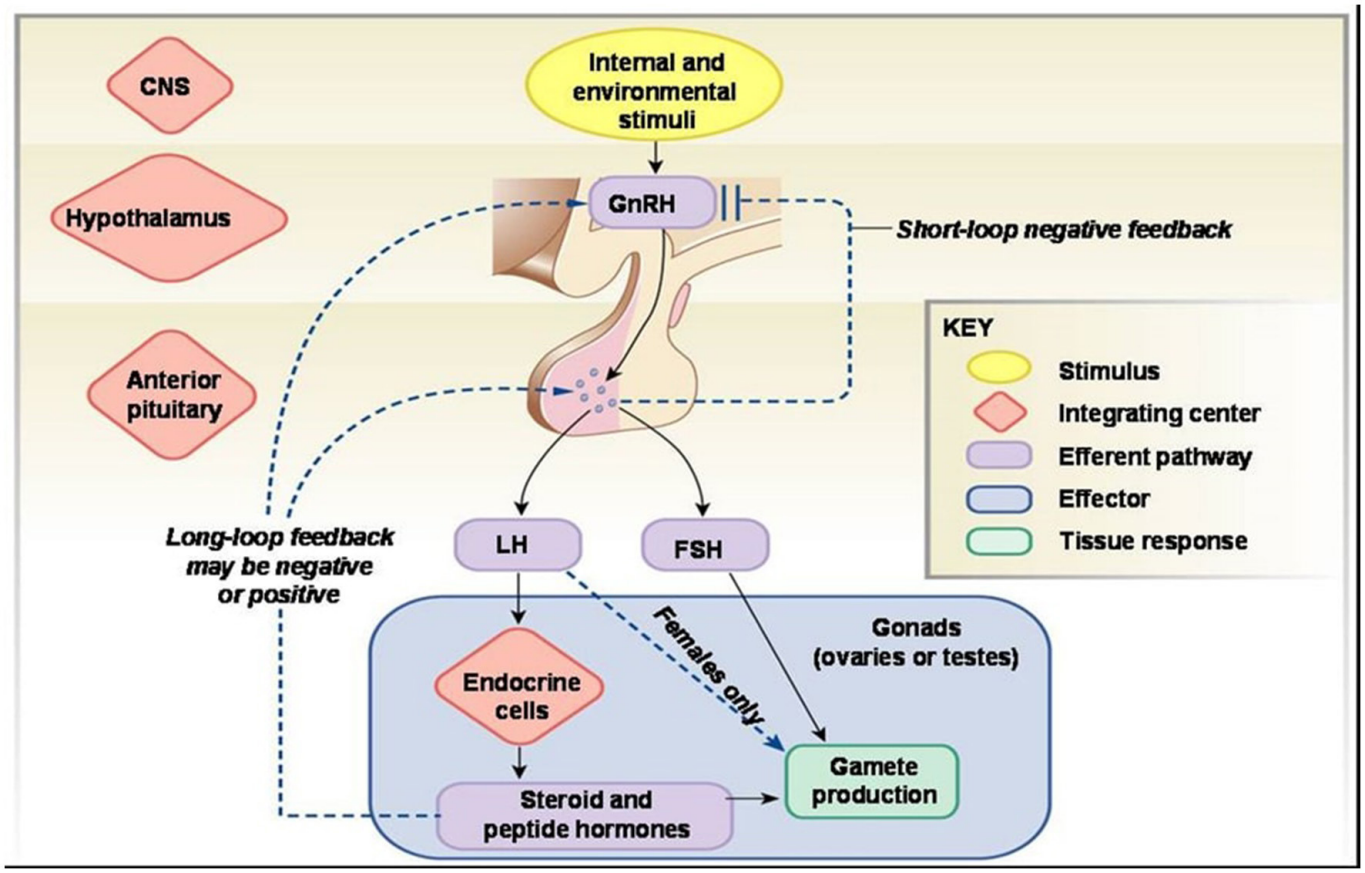
Hormonal mechanism of reproductive functions.^2^

Oocytes (egg cells) and somatic cells (granulosa cells, thecal cells, and stromal cells) interact in the ovary to direct the growth of follicles that contain oocytes, oocyte development, ovulation, and the establishment of the corpus luteum (the endocrine structure that forms from the ovarian follicle after ovulation and is required for establishing and maintaining pregnancy). Gonadotropin-releasing hormone (GnRH) pulses from the brain regulate the secretion of the hormones follicle-stimulating hormone (FSH) and luteinizing hormone (LH) from the anterior pituitary gland (Figure 6), which regulate many events in the adult ovary (37). Follicle-stimulating hormone (FSH) preferentially binds to granulosa cells, which stimulate the development of new follicles. The androgen- and estradiol-producing theca cells are affected by LH (36).

### The relationship between reproductive physiology and the brain: Light effect

The central nervous system (CNS) makes up the brain and spinal cord. It works with the peripheral nervous system (PNS) to regulate body functions. The brain is an extraordinary organ that regulates and controls functions related to the organism such as thought, memory, emotion, touch, motor skills, vision, breathing, temperature, starvation (38).

### Photoperiodism

In mammals, retinal photoperiodic information is found in the hypothalamus, the suprachiasmatic transfer to nuclei (SCN). SCN in the circadian hypothalamus is the primary pacemaker that produces rhythm. It manages the pacemakers in the light of the photoperiodic information it receives from the retina. Photoperiod is only essential in determining seasonality (39).

In mammals that reproduce seasonally, the day length or photoperiod is the primary regulator of reproduction. Photoperiodism consists of 2 parts photophase and scotophase. Photophase corresponds to the period when light is received, while scotophase corresponds to the period when there is no light (40). Decreased daylight or extended scotophase periods in sheep initiate seasonal reproduction, while an increase in day length or widening of photophase periods ends the breeding season. Short days in the winter season stimulatory on the neuroendocrine axis create a contradiction. When the day length increases again, the hypothalamic-pituitary-gonadal cycle begins to be suppressed again, and the sheep enter the anoestrus period. Sheep pregnant during the winter give birth to their offspring in spring or summer at the end of a 5-month gestation period, and the most suitable time for the offspring is established (41).

**Figure 7:**
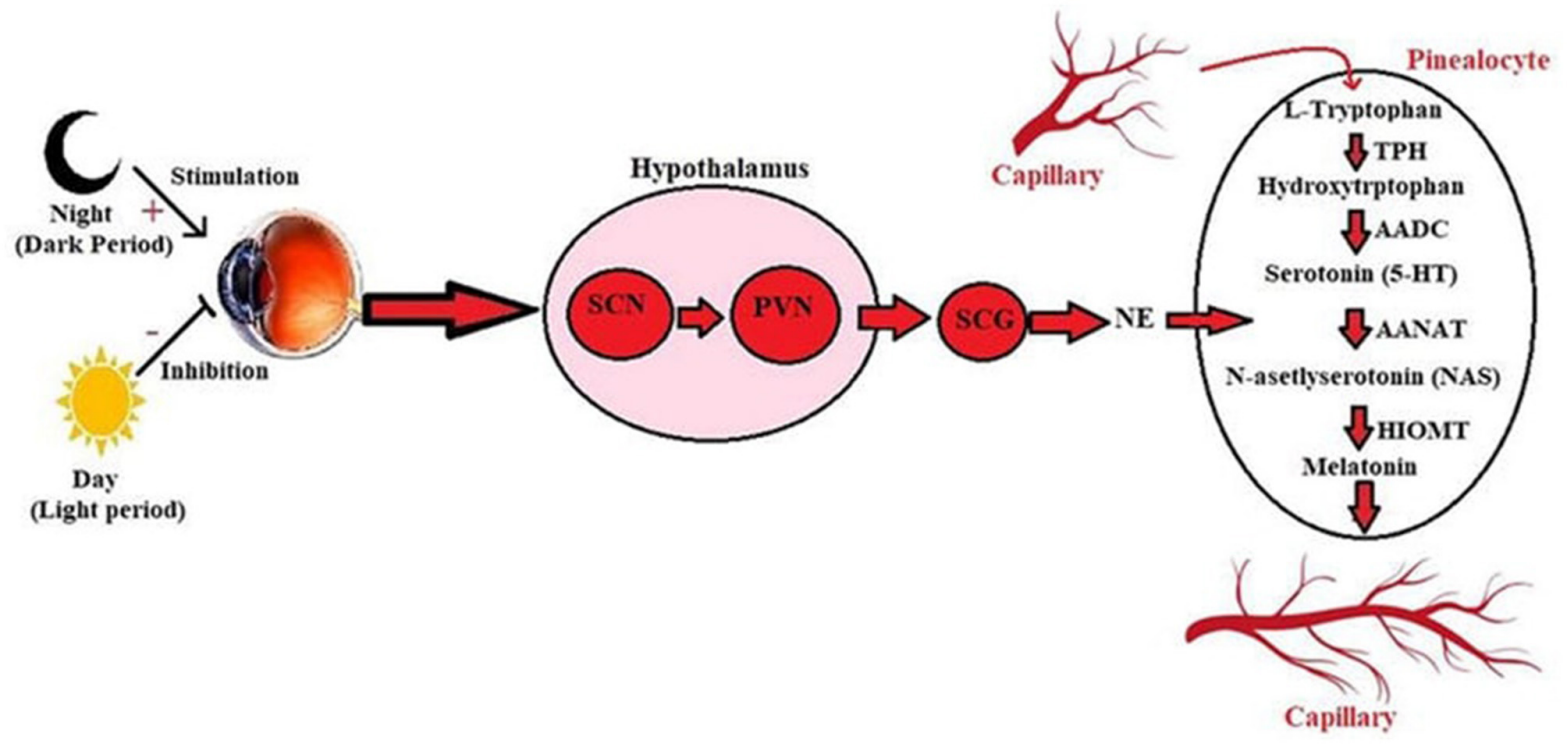
Synthesis of melatonin from tryptophan under the influence of light in the pineal gland (42). SCG, Superior cervical ganglion; SCN, Suprachiasmatic nucleus; NA, Noradrenaline; PVN, Paraventricular nucleus; AADC, Aromatic-L-amino-acid decarboxylase; AANAT, Arylalkylamine N-acetyltransferase; TPH, Hydroxytryptophan 5-hydroxylase; HIOMT, Hydroxy indole-O-methyltransferase; NAS, N-asetlyserotonin.

Circadian systems convert the light they receive into physiological processes. Light acts as a stimulator to trigger the response of the circadian clocks. Circadian clocks consist of a central clock in the SCN and clocks in peripheral organs. There is a pathway through which the connection is established between the retina of the eye and the SCN. This connection is made by the retinohypothalamic pathway (RHN). Activating in light, SCN inhibits neurons in the hypothalamus's paraventricular nucleus (PVN). The function of PVN is to release melatonin from the pineal gland. Inhibition of SCN on PVN indirectly inhibits melatonin secretion (Figure 7). Light inhibits melatonin secretion in this way. When the light disappears, and darkness occurs, this suppressive system disappears. Synthesized melatonin gives negative feedback on the pineal gland (43).

Melatonin is synthesized from the pineal gland and tryptophan in the retina of vertebrates. Although the synthesis of melatonin from both sources is light-based, their tasks are different from each other. Retinal melatonin regulates ocular physiology, such as retaining motor movements, regulating neurotransmitter release, and regulating neuronal electrical activities. Melatonin released from the pineal gland first passes into the blood vessel and cerebrospinal fluid. Melatonin is critical in managing central and peripheral tissues' physiological, biochemical, and behavioral processes. Tryptophan, the precursor of melatonin, is synthesized first. It is converted to 5- hydroxytryptophan by the enzyme hydroxylase and then converted to serotonin by 5-hydroxytryptophan decarboxylase. Serotonin is acetylated by an enzyme called arylalkylamine N -acetyltransferase (AANAT). In the final step, hydroxy- indole -O-methyl transferase converts N- acetylserotonin to melatonin. AANAT manages the rhythm of melatonin production (44). While AANAT expression and melatonin synthesis increase in dark environments, the level of this enzyme decreases in light exposure, and thus, melatonin synthesis decreases (45).

When animal species change, melatonin synthesized due to a dark environment is hypothalamic-pituitary-gonadal. The effects on the cycle also vary. While it effects negatively the reproductive system in animals with a long day cycle, on the contrary, it has a positive effect on the reproductive system in animals with a short day and long cycle. Sheep have a short day length. As the days get shorter, the photoreceptors' impulses in the retina go to the SCN *via* the retinohypothalamic pathway. SCN loses its inhibitory effect on the pineal gland. Removal of this inhibitory effect leads to melatonin secretion from the pineal gland. On the other hand, melatonin makes the GNRH level in the hypothalamus by binding to its specific receptors. At the same time, it increases the expression of FSH and LH synthesized from the anterior pituitary, thus increasing its level in plasma. These effects increase FSH and LH ovarian increase the cycling activity (46).

The pars tuberalis in the pituitary is one of the regions with the highest MT1 activity. It has a high affinity for thyrotropic cells in this region. With this effect, it controls the release of TSH. Therefore, while increasing melatonin increases TSH levels in sheep, decreasing melatonin levels causes a decrease in TSH levels. Eyes found in pars tuberalis when melatonin level decreases Increases the expression of absent 3 (Eya3). This causes an increase in TSH levels. The increased TSH goes to the nearest tissue and types two deiodinases. It increases the conversion of T4 to T3 by stimulating the (DIO2) enzyme. This, in turn, suppresses GnRH secretion. Thanks to this effect, sheep pass into the anoestrus period (46).

Have long day cycles animals increase their sexual cycles when the days start to get longer. Increasing the length of the day decreases the secretion of melatonin. In these animals, melatonin suppresses the reproductive system. Therefore, when the duration of the lights increases or the day increases, the level of melatonin in these animals decreases, and the hypothalamic-pituitary-gonadal cycle ceases from the suppressive effect of melatonin, and both GnRH and FSH, and LH levels begin to increase. At the same time, the perception of increasing day length also increases the TSH level. Increased TSH level is type 2 iodothyronine. While increasing the deiodinase (DIO2) level, the enzyme activity of type 2 iodothyronine deiodinase (DIO3) decreases. When the days decrease, the TSH level also decreases, and accordingly, the DIO2 enzyme activity decreases, while the DIO3 enzyme activity decreases, and as a result, the reproductive activity decreases (47).

Seasonal reproduction occurs with the effect of light taken from the retina in many animal species and is perceived from deep brain regions in birds. Also, the central circadian pacemaker is found not only on the SCN but also in the eye and pineal glands. In birds, light decreases in short days, photoreceptors in deep brain regions perceive this, and TSH decreases. While the decrease in TSH causes a decrease in DIO2 enzyme activity and increases enzyme activity, it also decreases reproductive activity. Likewise, as the number of long days increases, deep brain regions perceive this and increase the TSH level. While this decreases DIO3 activity, it increases DIO2 activity and stimulates reproductive activity (47).

### Pheromones, odor effect and a characteristic form of sexual behavior: Flehmen response

Pheromones are chemical signals that cause rapid behavioral responses in mammalian and non-mammalian species. The pheromones determined to be used in sexual communication by insects are named as “ectohormones”. The vomeronasal organ is the primary target of steroidal pheromones. Metabolites such as androgens, estrogens, glucocorticoids and vitamin D derivatives enter the vomeronasal duct and stimulate sensory neurons in the mammalian accessory olfactory bulbs (8).

**Figure 8:**
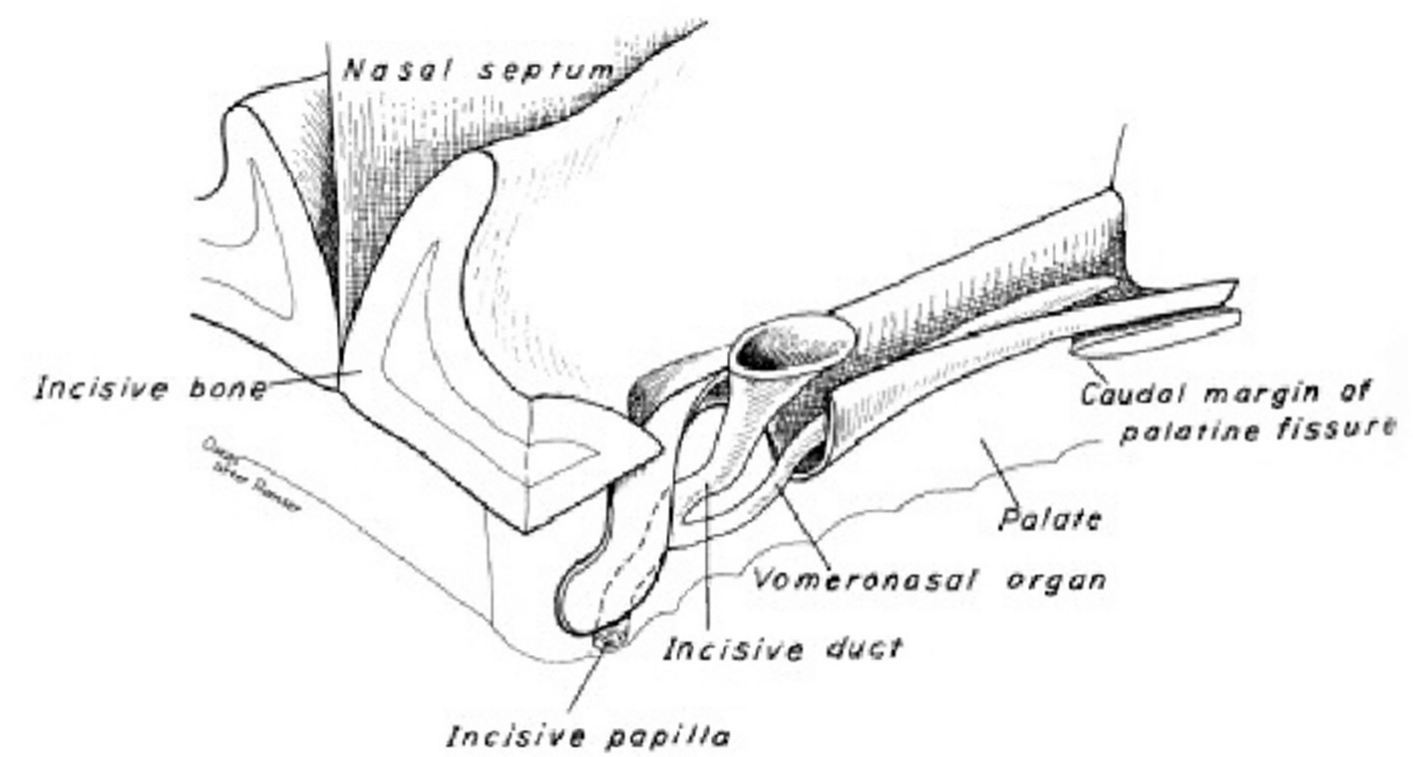
Dog vomeronasal organ (48).

The flehmen response or flehmen reaction is a behavior that lasts a few seconds and consists of an animal curling its upper lip upwards to show its front teeth while breathing, usually with its nostrils closed. For example, it can be done on an object or ground that attracts the animal, such as urine or feces, or by holding the head up. The flehmen response is observed in a wide range of mammals, including ungulates and felines. This behavior makes it easier for pheromones and other odors to reach the vomeronasal organ (Figure 8), located in the roof of the mouth, through a canal just behind the animal's front teeth (49).

### Heat effect

Heat is in a certain balance in all living things. The balance between heat lost and heat gained is known as thermal equilibrium. Excess heat in the body is tried to be removed from the body by various methods (convection, radiation, conduction, evaporation). The heat generated in the body is the result of metabolic activity in organs such as the heart, liver and muscle. In addition, the environment also has an effect on the formation of body temperature. Extremely hot and humid weather can cause hyperthermia in animals. In situations where the ambient temperature is too high, animals try to distribute their latent heat to the surrounding tissues as much as possible. However, if it is insufficient, animals direct blood to skin surfaces in order to cope with heat stress. In addition, sweating and accelerating breathing also play a role in removing heat. Vasodilation is very important in removing body heat. The temperature increase that occurs is perceived by the central nervous system, and at the same time, vasodilation can be formed in the body with the sensations of thermoreceptors (50).

Heat, which is so important for living things, is regulated from the preoptic area of the hypothalamus. This region detects the increased body temperature with all peripheral and central signals. Especially the medial preoptic area plays a role in the formation of sexual and parental behaviors. It has a role in many behaviors such as sexual behavior, copulation, intromission and ejaculation in males. In females, it is known that the preoptic area regulates effects such as baby care and nest building (51). High temperature conditions also negatively affect reproductive functions by causing dysfunctions in the hypothalamic-pituitary-gonadal axis. It shows these effects through FSH, LH, and GnRH (52).

Sexual behaviors are directly related to the steroid hormones that cause sexual arousal in the brain. The fact that this process is more effective in terms of sexual experience paves the way for animals to establish relationships between stimuli and behaviors that cause different sexual responses (53). Table 1 summarizes the sexual behaviors specific to various animal species.

**Table 1:** Sexual behaviors specific to different animal species.

**Animal Models**		**Sexual behavior**	**References**
Laboratory Animals	Rat (female)	Paracopulatory behavior: hopping (short jumps with all four legs off of the ground) and darting (short and sudden runaway movements, in which she presents her body to the male)	(54)
	Rabbit (male)	Sexual aggressiveness such as grasping and kicking	(55)
Pet Animals	Bitch	Interaction with the male (mounts on the male), frequent urination, perineum movements, increased interest in male urine and anal sac odors	(56)
	Tomcat	Olfactory cue in female urine, sniffing, holding the queen neck	(57)
Farm Animals	Stallion	Tactile investigation, biting and nuzzling, flehmen, vocalizations	(58)
	Mare	Ears back, urination, exposing the perineal region, clitoral winking	(59)
	Buck	Kicking, vocalization, anogenital sniffing, flehmen, nudging, tongue-lapping, enurination, tail straight	(60)
	Ram	sniffing females genitals, flehmen, kicking, nudging, vocalization	(61)

It is important to eliminate some of the problems that arise in terms of the sustainability of the sexual life of animals. In the Tables 2, 3, some clinical signs regarding sexual dysfunctions and the active substances used against some neurological diseases that cause sexual dysfunction are mentioned.

**Table 2:** Sexual dysfunctions caused by neurological diseases.

**Disease**	**Clinical sign**	**References**
Kallmann syndrome	Hypogonadotropic hypogonadism and anosmia or hyposmia	(62)
Multiple sclerosis (MS)	Adverse effects on sexual response and sexual functions	(63)
Spinal cord injury (SCI)	Erectile dysfunction, adverse effects on ejaculatory process and orgasm, and male reproductive potential.	(64)
Prostatectomy or surgery for bladder cancer	Erectile dysfunction, problems with libido, arousal, orgasm, and dyspareunia	(65)
Diabetic peripheral neuropathy	Erectile dysfunction	(66)
Parkinson's disease	Reluctance to engage in sex, problems with ejaculation, lubrication and urinary incontinence	(67)
Epilepsy	Sexual dysfunction	(68)

**Table 3:** Therapeutic substances used in the treatment of some neurological diseases characterized by sexual dysfunction.

**Neurological disease**	**Drug used in treatment**	**References**
Multiple sclerosis (MS)	Sildenafil	(69)
Spinal cord injury (SCI)	Sildenafil	(70)
Epilepsy	Lamotrigine	(71)
Parkinson's disease	Apomorphine	(72)

Sexual behavior disorders related to the clinical reflections of some neurological diseases are at a level that can respond to treatment. Therefore, research on the sexual behavior of animals gains meaning in terms of neuroscience, as highlighted in Tables 2, 3.

## Conclusion

Sexual behaviors differ between animal species. Genetic, endocrinological, anatomical, physiological, and environmental factors play a role in this difference. The communication and interaction between the brain and the gonads constitute a fundamental axis for reproduction. In terms of a sustainable livestock and a healthy ecosystem, it is necessary to interpret the sexual behavior and reproductive activities of animals effectively.

## Author contributions

ST, EA, and AÖ contributed to conception, design of the study, and wrote sections of the manuscript. AÖ wrote the first draft of the manuscript. All authors contributed to manuscript revision, read, and approved the submitted version.

## Footnotes

^1^ https://www.msdvetmanual.com/horse-owners/reproductive-disorders-of-horses/the-gonads-and-genital-tract-of-horses

^2^ https://www.austincc.edu/apreview/PhysText/Reproductive.html
